# Immunosuppression in Honeybee Queens by the Neonicotinoids Thiacloprid and Clothianidin

**DOI:** 10.1038/s41598-017-04734-1

**Published:** 2017-07-05

**Authors:** Annely Brandt, Katharina Grikscheit, Reinhold Siede, Robert Grosse, Marina Doris Meixner, Ralph Büchler

**Affiliations:** 1 LLH Bee Institute, Erlenstr. 9, 35274 Kirchhain, Germany; 2 Institute of Pharmacology, Biochemical-Pharmacological Center (BPC), University of Marburg, 35032 Marburg, Germany

## Abstract

Queen health is crucial to colony survival of honeybees, since reproduction and colony growth rely solely on the queen. Queen failure is considered a relevant cause of colony losses, yet few data exist concerning effects of environmental stressors on queens. Here we demonstrate for the first time that exposure to field-realistic concentrations of neonicotinoid pesticides can severely affect the immunocompetence of queens of western honeybees (*Apis mellifera* L.). In young queens exposed to thiacloprid (200 µg/l or 2000 µg/l) or clothianidin (10 µg/l or 50 µg/l), the total hemocyte number and the proportion of active, differentiated hemocytes was significantly reduced. Moreover, functional aspects of the immune defence namely the wound healing/melanisation response, as well as the antimicrobial activity of the hemolymph were impaired. Our results demonstrate that neonicotinoid insecticides can negatively affect the immunocompetence of queens, possibly leading to an impaired disease resistance capacity.

## Introduction

Honeybees are highly eusocial insects that build colonies of several thousand individuals which contain only one fertile female, the queen^1^. This queen is responsible for all egg laying and brood production within the colony; consequently, her integrity and health is crucial for the colony’s performance and survival, and any impairment can result in adverse effects on colony fitness. In the worst case, if the workers are unable to replace a failing queen, the colony will perish^2–4^. Recently, queen failure has been proposed as an important driver of honeybee colony mortality^2, 4–6^. While the natural lifespan of a honeybee queen is two to four years^1^, recent reports from the U.S.A. show high rates of early queen failure, with 50% or more being replaced in colonies within the first six months^2, 3^. This extremely high rate of queen failure coincides with high mortality rates of colonies in the U.S.A., where in some years more than 50% of colonies are dying^2, 7^.

Several stress factors are suspected to negatively affect survival of honeybee colonies. Parasites and pathogens are among the main factors^4^, but diet quantity, quality, and diversity^8–10^ as well as exposure to pesticides may also affect colony survival^5, 6, 11^. In particular, the widespread application of neonicotinoid insecticides^12–18^, has been suspected to represent a major threat to honeybee survival^11, 19–22^. Neonicotinoids are neurotoxins that act as agonists of the nicotinic acetylcholine receptor. They disrupt the neuronal cholinergic signal transduction, leading to abnormal behaviour, immobility and death of target pests^12, 23, 24^. Frequently, non-target insects like honeybees also come into contact with these systemic insecticides^21^. Exposure of bees to neonicotinoids is mostly through ingestion of residues in the pollen and nectar of contaminated plants^11, 19^. These pesticide residues are taken by the forager bees to their colonies and remain stored in beebread or honey until they are fed to larvae, workers, drones, or the queen^4, 25^.

The study of lethal and sub-lethal effects of neonicotinoid pesticides on social bees has largely focused on worker bees and, to a lesser extent, on overall colony function^18, 25–28^. Although reports of colony losses due to high rates of queen failure exist, only few studies address direct or indirect physiological effects in queens caused by pesticide exposure^2, 5, 29, 30^. Most studies focused on only three neonicotinoid insecticides, clothianidin, imidacloprid, and thiamethoxam, which are currently subject to a moratorium in the European Union^31^. Here, we also include thiacloprid, a cyano-substituted neonicotinoid which is considered as non-harmful for bees due to its much lower acute toxicity compared to other neonicotinoids. Thiacloprid is widely used in agriculture, for instance as spray application in fruit trees or directly into flowering oil seed rape during bee flight. It is the most abundant insecticide in beebread samples and has been detected in more than 60% of the samples analysed in the German Bee Monitoring Project^32^.

A strong immune defence is vital for honeybee health and colony survival. The strength of the individual immune defence depends on internal factors such as the nutritional state, the age, and the caste affiliation of the exposed individuals^10, 33–37^. In addition, the invasive ectoparasite *Varroa destructor* ^38^ impairs the immune defence of honeybees by reducing expression of immune-relevant genes and boosting viral replication, thereby affecting lifespan and disease resistance^11, 39–41^. The individual immunocompetence can also be weakened by environmental factors like pesticides that may render honeybees more vulnerable to parasites and pathogens^41–44^.

The exposure to pesticides is often associated with an increased pathogenic load, including the prevalent gut-parasite *Nosema* spp. and viruses typically associated with *V*. *destructor*, such as deformed wing virus (DWV)^8, 11, 43, 45, 46^. Neonicotinoids have been shown to affect the individual immunocompetence of honeybee workers. They negatively modulate NF-κB immune signalling and promote the replication of DWV^44^. Neonicotinoids have been shown to reduce hemocyte density as well as functional aspects of insect immunity as the melanisation of foreign objects, and the antimicrobial activity of the hemolymph^47^ in worker bees. However, there is only little information available about the individual immunity of honeybee queens. In queens, phenoloxidase (PO) enzyme levels continuously increase with age and reach levels twice as high as those found in workers^48^. Gätschenberger and colleagues^49^ examined the antimicrobial defence systems of queens and found that reactions of young queens to bacterial immune challenges resemble those of worker bees. In this study we examine the effects of pesticide exposure on young queens. Our main question is whether general immune defence mechanisms are affected by sublethal concentrations of the neonicotinoids thiacloprid or clothianidin. Since disease resistance is difficult to measure directly^33^, we selected established functional parameters of immunity to analyse honeybee immunocompetence, namely total and differential hemocyte counts, melanisation response, and antimicrobial activity of the hemolymph.

## Results

### Sublethal effects and exposure

To date, acute toxicity tests are solely performed with worker bees^﻿5, 25^. To determine whether the dosages of neonicotinoids used in the immune assays are indeed sublethal to queens, the number of dead queens was recorded after seven days of exposure. We found no significant difference between the treatment groups regarding the survival of the queens (Supplementary Fig. S1, Supplementary Table S2). Thiacloprid exposure had no lethal effect on worker bees. However, there was a significant effect of clothianidin on worker bee mortality (χ^2^-test; p = 0.009). Hence, the highest concentration of clothianidin (200 µg/l) was excluded from immune tests.

To estimate the exposure level to neonicotinoids, the food consumption was measured. We found no significant difference between the treatment groups regarding the consumption of sugar solution or pollen (Supplementary Fig. S2, Supplementary Table S2). Moreover, the behaviour of the bees was observed regularly. When the food was changed, the queens were surrounded by attending workers and fed via trophallaxis. During these short observations, we never saw a queen directly feeding at the syringe containing the spiked sugar solution.

The wellbeing of a queen depends on the care and the adequate supply with food (royal jelly and honey^1^) by the attending worker bees. However, neonicotinoids have been shown to reduce the size of the hypopharyngeal gland (HPG) of nurse bees. To examine, whether thiacloprid or clothianidin affected the HPG size of the attending worker bees in our experiments, the acinus diameter of age defined worker bees were measured. Interestingly, no significant difference was found in the HPG size of worker bees after seven days of exposure in a cage, attending to a queen (Supplementary Fig. S3, Supplementary Table S2; KWT, p > 0.05).

### Total and differential hemocyte counts

**Figure 1:**
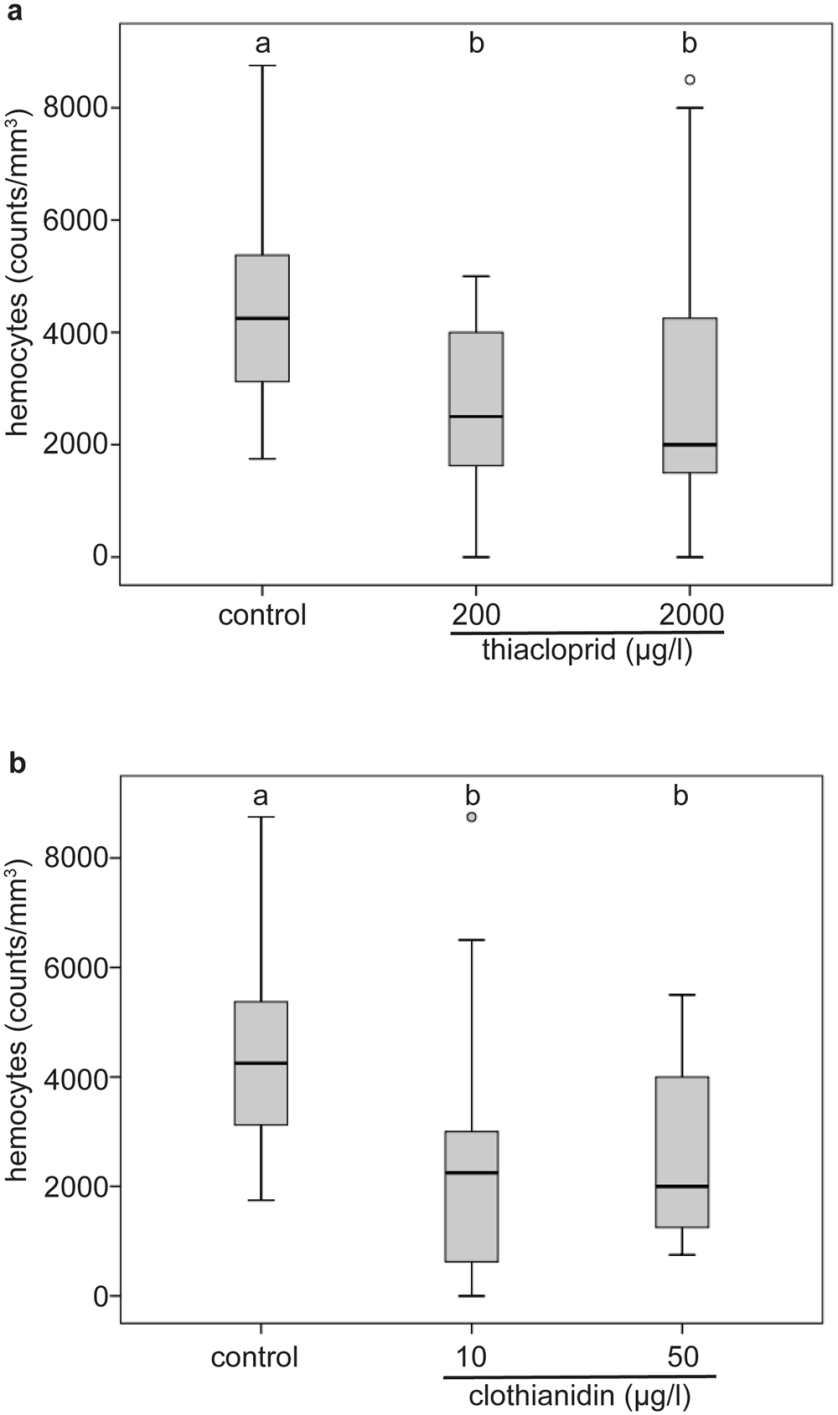
Exposure to thiacloprid or clothianidin reduced total hemocyte counts. The seven day-treatment of young honeybee queen with thiacloprid ((**a**); control: n = 19, 200 µg/l: n = 16, 2000 µg/l: n = 19), or clothianidin ((**b**); control: n = 19; 10 µg/l: n = 15; 50 µg/l: n = 14) reduced the total hemocyte counts compared to control queens. Boxes show 1st and 3rd interquartile range with black lines denoting medians. Whiskers encompass 95% of the individuals, beyond which outliers (circles) reside. Treatments with different letters differ significantly from each other.

Exposure to relevant concentrations of both neonicotinoids significantly reduced the total hemocyte counts of young queens (Fig. 1a,b). The median total hemocyte counts of thiacloprid treated queens were lower than those of control queens (Fig. 1a, Kruskal–Wallis test (KWT), p = 0.011). Untreated control queens displayed a higher hemocyte density than queens treated with 200 µg/l thiacloprid (59% of control value; Supplementary Table S1; MWU, p = 0.022; control: median = 4250 hemocytes/µl (h/µl), n = 19; 200 µg/l thiacloprid: median = 2500 h/µl, n = 16), or treated with 2000 µg/l thiacloprid (47% of control value; MWU, p = 0.024, median = 2000 h/µl, n = 19).

Total hemocyte counts of queens treated with clothianidin were lower than in control queens (Fig. 1b, KWT, p = 0.0005), with control queens displaying a higher hemocyte density than queens treated with 10 µg/l clothianidin (53% of control value; MWU, p = 0.015; control: median = 4250 h/µl, n = 19; 10 µg/l clothianidin: median = 2250 h/µl, n = 15), or treated with 50 µg/l clothianidin (47% of control value; median = 2000 h/µl, n = 14, MWU, p = 0.014).

**Figure 2:**
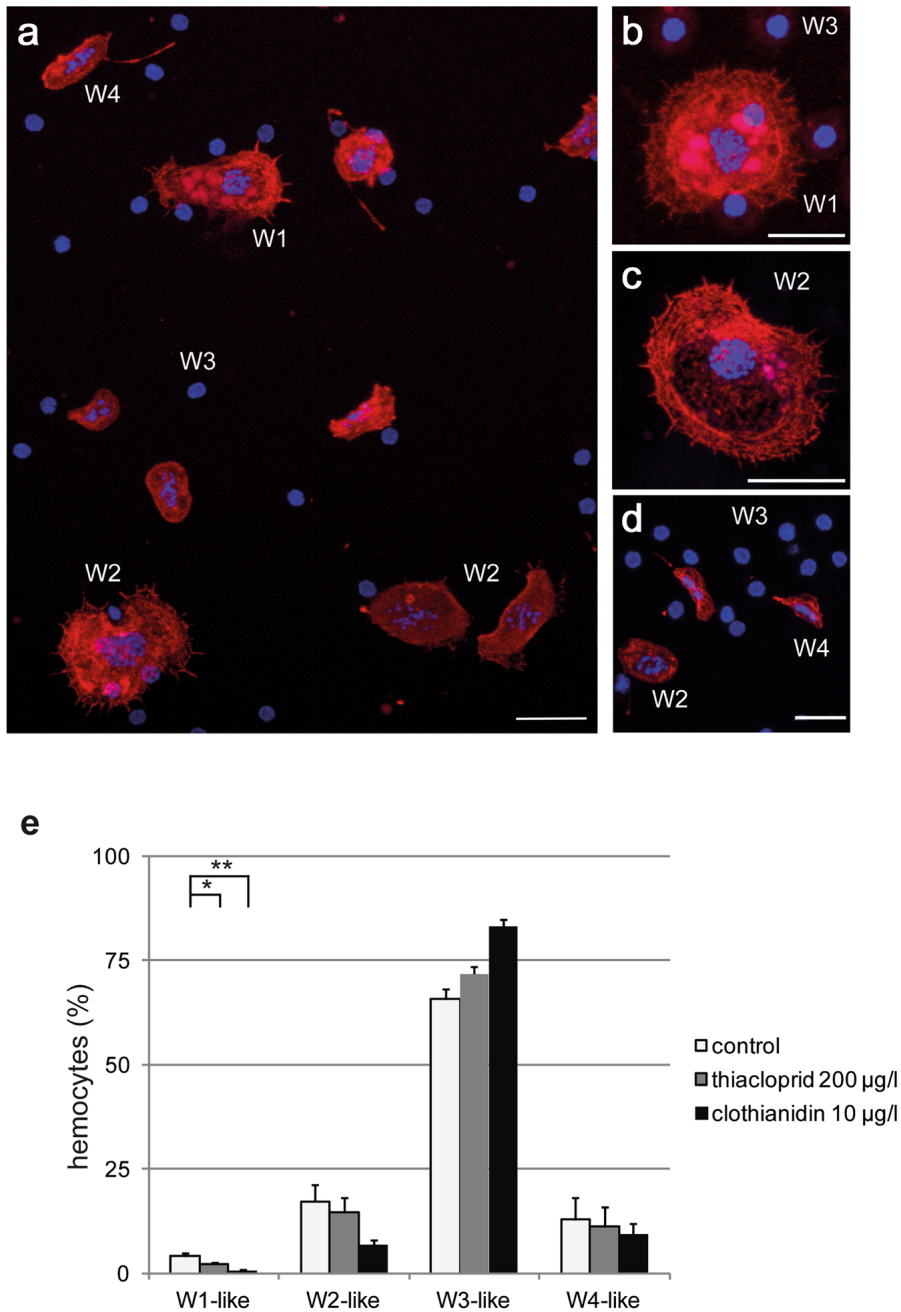
Exposure to neonicotinoids differentially affected hemocyte subclasses. (**a**–**e**) Queen hemocytes showing staining of the filamentous actin cytoskeleton (red: phalloidin-staining) and nuclear DNA (blue: DAPI-staining). (**a**) Compound image. W1-like hemocytes contained vesicle-like structures and showed extreme cellular spreading, lammelipodia formation, and filopodia-like structures with a well developed cytoskeleton marked by intensive f-actin staining (**a**,**b**; W1). W2-like hemocytes contained no vesicle-like structures, but also showed spreading and strong f-actin staining (**a**,**c;** W2). The nuclei of W1- or W2-like hemocytes were large and contained decondensed chromatin. W3-like hemocytes were oval/round and had only very weak f-actin staining. Nuclear chromatin of W3 like cells was highly condensed (**a**,**b**,**d**; W3). W4-like hemocytes had a spindle-shaped morphology and showed intensive f-actin staining of the cytoskeleton and elongated nuclei (**a**,**d**; W4). (**e**) Thiacloprid (200 µg/l) or clothianidin (10 µg/l) treatment affected the composition of the hemocyte population (8 to 9 individuals per treatment group, at least 350 cells per individual counted). Thiacloprid or clothianidin exposure significantly reduced the number of W1-type hemocytes. Clothianidin treated queens had a lower percentage of W2-like cells and a higher percentage of W3-like cells compared to control queens (error bars = s.e.m; *p ≤ 0.05; **p ≤ 0.01; scale bar 10 µm).

To evaluate changes in the composition of subclasses in the hemocyte population, the cells were classified using the morphological characteristics described by Negri *et al*.^36, 50^, supported by phalloidin staining of the actin cytoskeleton and DAPI staining of the nuclear DNA (Fig. 2a–d). W1-like hemocytes^50^ contained a large oval or irregular shaped nucleus and prominent vesicle-like structures. These granulocyte-like hemocytes^36, 50^ had a well-developed actin cytoskeleton marked by intensive f-actin staining, and showed extreme cellular spreading as indicated by lammelipodia and numerous filopodia-like structures (Fig. 2a,b). The hemocytes of the subclass W2 had large, round or oval shaped nuclei with decondensed chromatin, but did not contain vesicle-like structures (Fig. 2a,c,d). Some W2-like hemocytes showed spreading with an intensively stained actin cytoskeleton, lammelipodia formation, and filopodia-like structures (Fig. 2c). Hemocytes of the W3-like subclass were the most abundant ones. They were small, of oval or round shape and contained small nuclei with highly condensed chromatin. In contrast to the other cell types, W3-like cells showed no spreading and had only very weak f-actin staining (Fig. 2a,b,d). Queen hemocytes classified as W4-like had a spindle-shaped morphology with well-developed actin cytoskeleton and small, elongated or irregular shaped nuclei (Fig. 2a,d).

Pesticide treatment differentially affected the relative abundance of hemocyte subclasses. Exposure to 200 µg/l thiacloprid and 10 µg/l clothianidin reduced the percentage of W1-like cells in treated queens compared to controls (Fig. 2e, KWT, p = 0.0005; MWU, control vs. 200 µg/l thiacloprid: p = 0.002, control vs. 10 µg/l clothianidin: p = 0.034; control: average = 3.356%, n = 9; 200 µg/l thiacloprid: average = 1.980%, n = 9; clothianidin: average = 0.394%, n = 8). Exposure to 10 µg/l clothianidin produced a detectable reduction of W2-like hemocytes (Fig. 2e, KWT, p = 0.0384; MWU, control vs. 200 µg/l thiacloprid: p > 0.05, control vs. clothianidin: p = 0.06; control: average = 14.17%; 200 µg/l thiacloprid: average = 7.97%; clothianidin: average = 6.67%). Clothianidin treated queens showed a relative increase of undifferentiated, W3-like hemocytes (Fig. 2e, KWT, p = 0.0408; MWU, control vs. 200 µg/l thiacloprid: p > 0.05, control vs. clothianidin: p = 0.077; control: average = 69.06%; 200 µg/l thiacloprid: average = 72.73%; clothianidin: average = 83.27%). There was no significant difference between the treatment groups regarding the spindle-shaped W4-like hemocytes.

### Melanisation

**Figure 3:**
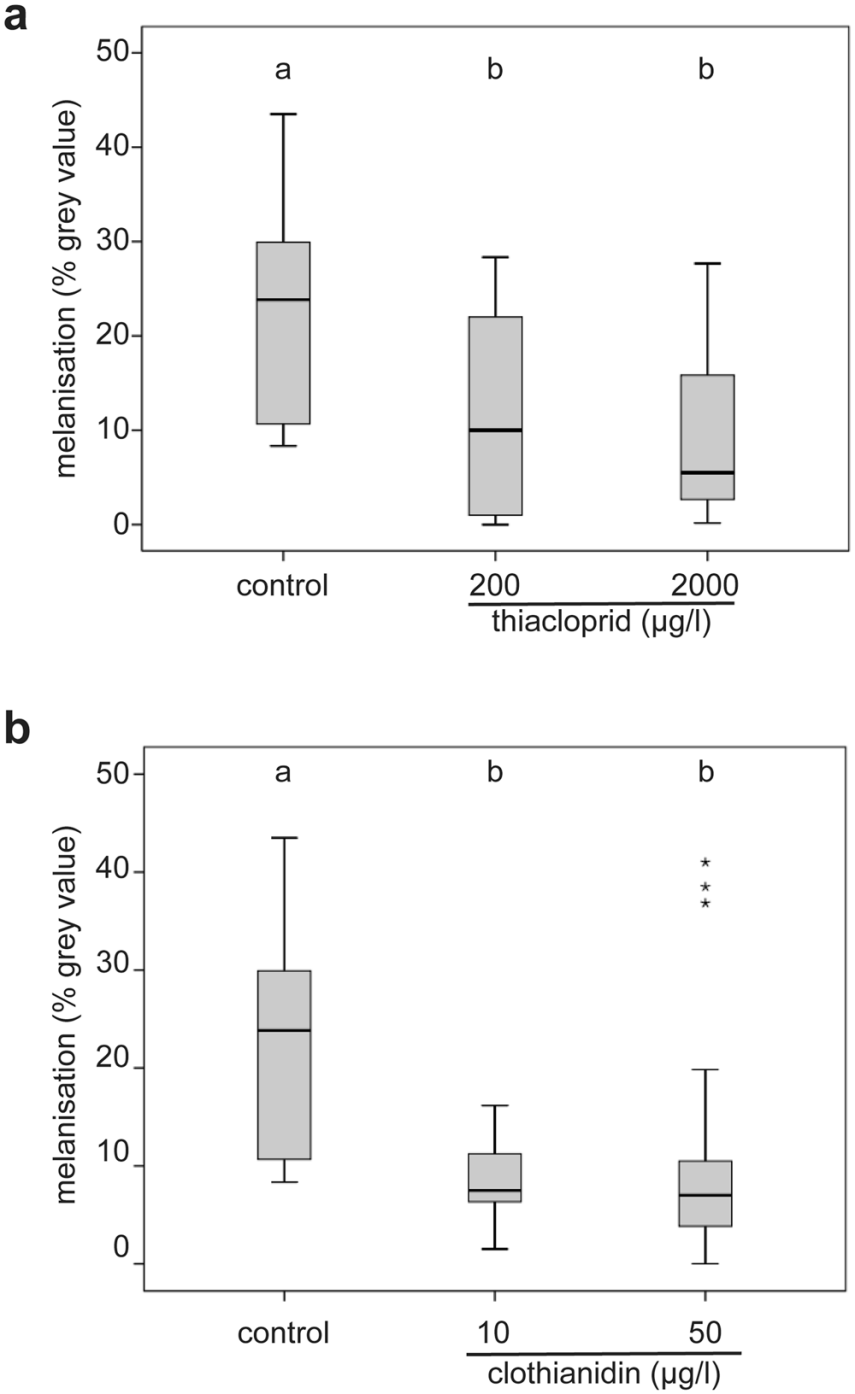
Exposure to thiacloprid or clothianidin reduced melanisation response. The seven day-exposure of young honeybee queens to thiacloprid ((**a**); control: n = 20, 200 µg/l: n = 17, 2000 µg/l: n = 15), or clothianidin ((**b**); control: n = 20; 10 µg/l: n = 19; 50 µg/l: n = 18) reduced the melanisation of an implanted nylon filament compared to control queens. Boxes show 1st and 3rd interquartile range with black lines denoting medians. Whiskers encompass 95% of the individuals, beyond which outliers (asterisks) reside. Treatments with different letters differ significantly from each other.

Compared to control queens, the melanisation response of queens treated with neonicotinoids was significantly reduced at all concentrations tested (Fig. 3). The melanisation in thiacloprid treated queens was reduced to 41% (200 µg/l) or 23% (2000 µg/l) of the control value (Fig. 3a, KWT, p = 0.001; MWU, control vs. 200 µg/l thiacloprid: p = 0.016, control vs. 2000 µg/l thiacloprid: p = 0,003; control: median = 23.83% grey value (gv), n = 20; 200 µg/l thiacloprid: median = 10% gv, n = 17; 2000 µg/l thiacloprid: median = 5.5% gv, n = 15). The melanisation in clothianidin treated queens was reduced to 31% (10 µg/l) or 29% (50 µg/l) of the control value (Fig. 3b, KWT, p < 0.0001). Control queens showed a stronger melanisation than queens treated with clothianidin (Mann–Whitney U test (MWU), control vs. 10 µg/l clothianidin: p < 0.0001, control vs. 50 µg/l clothianidin: p = 0.002; control: median = 23.83% gv, n = 20; 10 µg/l clothianidin: median = 7.5% gv, n = 19; 50 µg/l clothianidin: median = 7.00% gv, n = 18).

### Antimicrobial activity of the hemolymph

**Figure 4:**
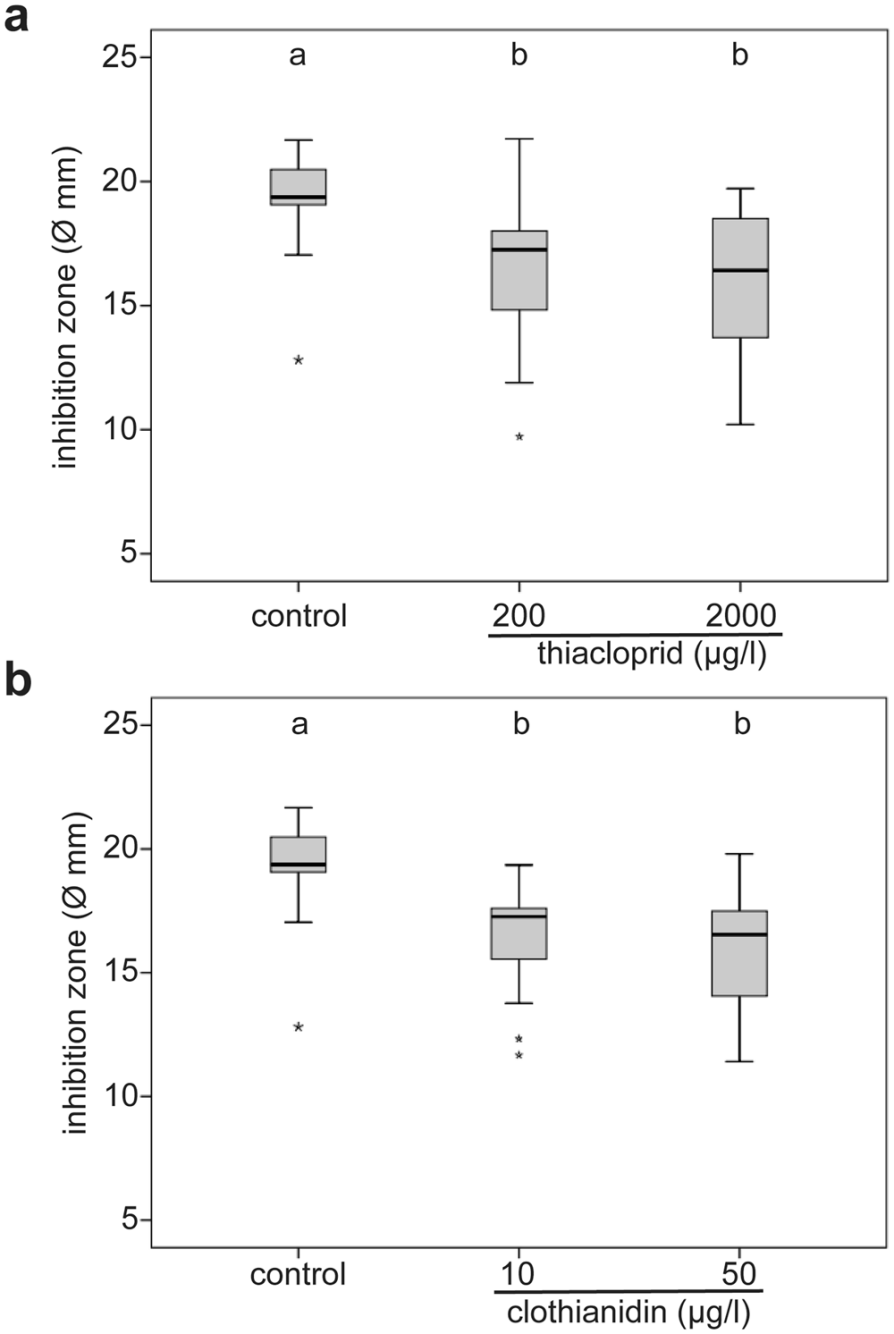
Neonicotinoid exposure reduced antimicrobial activity of hemolymph. The hemolymph inhibited the growth of grampostive bacteria (*Micrococcus flavus*) on agar plates. The seven day-treatment with thiacloprid ((**a**), control: n = 15: 200 µg/l: n = 16; 2000 µg/l: n = 15) or clothianidin ((**b**), control: n = 15: 10 µg/l: n = 14; 200 µg/l: n = 14) reduced the antimicrobial activity of the hemolymph, the diameter of the inhibition zones being smaller than in control queens. Boxes show 1st and 3rd interquartile range with black lines denoting medians. Whiskers encompass 95% of the individuals, beyond which outliers reside (asterisks). Significant differences indicated with letters.

The antimicrobial activity of the hemolymph, measured as the size of the inhibition zones, was significantly reduced in queens treated with all concentrations of thiacloprid or clothianidin compared to control queens (Fig. 4, KWT, p = 0.002). Inhibition zones of queens exposed to thiacloprid were significantly smaller than in control queens (89% of control value; MWU, p = 0.008; control: median = 19.37 mm, n = 15; 200 µg/l thiacloprid: median = 17.25 mm, n = 16), or treated with 2000 µg/l thiacloprid (84% of control value; MWU, p = 0.003, median = 16.42 mm, n = 15). Inhibition zones of queens treated with clothianidin were significantly smaller than in control queens (Fig. 4b, KWT, p < 0.001), with control queens displaying a larger inhibition zone than queens treated with 10 µg/l clothianidin (89% of control value; MWU, p < 0.001, median = 17.26 mm, n = 14), or treated with 50 µg/l clothianidin (85% of control value; MWU, p < 0.001, median = 16.54 mm, n = 14).

## Discussion

In this paper we report immunosuppressive effects of two neonicotinoid pesticides on general immune parameters of honeybee queens. We employed a broad array of methods to investigate the immune defence competence of queens: total and differential hemocyte counts, wound healing/melanisation, and antimicrobial activity of the hemolymph after immune stimulation. Our results indicate that the tested aspects of individual immunity are negatively affected by sublethal, environmentally relevant concentrations of neonicotinoids in newly emerged honeybee queens.

Hemocytes are the key components of insect cellular immune defence. They are responsible for phagocytosis or encapsulation of pathogens and for the closure of wounds^36, 51^. The mechanisms of cellular immunity in honeybees are not yet completely understood, mostly due to limited information about the cellular subtypes, their functions and abundances^50, 52, 53^. We found an overall reduction of hemocyte density at all concentrations tested. Based on morphological characterisation, we also observed changes in the abundance of subclasses in the population of hemocytes in clothiandin exposed queens, which may indicate a severe interference of neonicotinoid treatment with cellular immunity.

The staining of the actin cytoskeleton by phalloidin together with nuclear staining can be used as a valuable tool for hemocyte characterization. Granulocytes are the first type of hemocyte to attach to a foreign object during the encapsulation response^50^. We found a significant reduction in the number of granulocyte-like hemocytes in queens exposed to clothianidin. As in workers^50^, these granulocyte-like cells showed extreme spreading with extensive filopodia and lammellipodia formation and contained numerous vesicle-like structures which may indicate their involvement in phagocytosis^36, 50^. The reduction of granulocyte-like hemocytes may be particularly severe, since this type of active, differentiated hemocyte is considered responsible for the elimination of pathogens and is the first cell type to initiate the encapsulation process^36, 54^. In addition, W2-like hemocytes tended to be reduced in queens treated with clothianidin. The W2 hemocytes have been shown to be the second cell type which adheres to foreign surfaces, a specific function of this cell type has not yet been described^50^.

Hemocytes of the types W3 and W4 are normally freely floating in the hemolymph and interact with adherent granulocytes to form cell agglomerations^36^. We found a relative increase of W3-like cells in queens exposed to clothianidin. The overall number of W3- or W4-like hemocytes may be underestimated in our study, since most likely not all of them adhered to the surface of the glass cover slip^36^. In conclusion, an altered hemocyte density together with a reduction in specific subclasses of active, differentiated hemocytes caused by neonicotinoid exposure could likely impair the cellular immune defence, hence increasing a queen´s susceptibility towards pathogens. Nevertheless, honeybee hemocyte typing remains complex and it is difficult to compare the results obtained by different methods^50, 52, 53^. More detailed investigations are necessary to bring light into the process of hemocyte differentiation in honeybees, combining the development of reliable cell markers and time-lapse microscopy.

A central immune defence mechanism mediated by hemocytes is the encapsulation and melanisation of intruding pathogens and the closure of wounds. The melanisation reaction is catalysed by the enzyme phenoloxidase whose precursor prophenoloxidase is produced by hemocytes^51^. We observed a significantly reduced melanisation after treatment with neonicotinoids in all substances and concentrations tested. The ability to melanise and encapsulate a foreign object is positively correlated with resistance to viral infections, parasitoids, and parasites. Wound closure involves similar mechanisms as encapsulation and melanisation and is important for reducing virus transfer between bees^51, 55^. In addition, we showed that exposure to thiacloprid or clothianidin significantly reduced the antimicrobial activity of the hemolymph. The antimicrobial activity depends on the amount of antimicrobial peptides in the hemolymph, which are produced by hemocytes or fat body cells^55^. Together with the observed decrease in the melanisation response and changes in hemocyte composition and density, our findings may be interpreted as impairments of immune defence and disease resistance capacity of honeybee queens during exposure to neonicotinoids.

In our cage experiments, exposure to thiacloprid and clothianidin significantly affected the immunocompetence of young queens, even in sublethal concentrations as low as those reported from samples collected by honeybees. The thiacloprid or clothianidin concentrations fed over a period of seven days showed no lethal effects on queens and were therefore considered as sublethal for queens. Only the extremely high concentration of 200 µg/l clothianidin increased the mortality of attending worker bees and was therefore excluded from further analysis. According to Sanchez-Bayo and Goka^56^, mean thiacloprid residues are 75.1 µg/kg (max.: 1002 µg/kg, mean prevalence of 17.7%) in pollen, and 6.5 µg/kg in honey (max.: 208 µg/kg, 64% prevalence)^56^. Mean clothianidin residues in pollen were 9.4 µg/kg (max.: 41.2 µg/kg, mean prevalence of 11%) and in honey 1.9 µg/kg (max.: 10.1 µg/kg, mean prevalence of 17%). In the German bee monitoring, the highest observed concentrations of thiacloprid in beebread samples were 498 µg/kg^32^. Thiacloprid is frequently detected in honey samples, up to concentrations of 200 µg/kg^4, 25^.

In a colony the queen is constantly surrounded by attending worker bees which take care of her wellbeing and constantly supply her with high-quality food such as royal jelly. A queen can survive without worker bees for only a few days. However, this is a non-natural situation with unknown effects on the immune status of a queen. To approximate the natural situation in a laboratory experiment, ten attending bees were placed into each cage to provide for the queen. However, this makes it difficult to discern between the direct effects of neonicotinoids on the immune system of queens and indirect effects that may come from affected worker bees. In the concentrations used in immune assays, the mortality of queens or workers was not increased. Moreover, food consumption of sugar solution or pollen was comparable in all treatment groups, indicating normal feeding behaviour. Interestingly, in the presence of a queen, the neonicotinoids had no significant effect on the size of the HPG compared to controls. The mechanism by which xenobiotics like neonicotionoids, fungicides or varroacides affect HPG size is still unknown. Our findings may imply that the stimulus provided by the queen may counteract the HPG-reducing effects described for neonicotinoids. Although we cannot rule out any possible indirect effect, when considering the normal survival rate, the normal food consumption and the normal size of the HPGs together, we have no indications that our experimental queens may have suffered from a lack of care or feeding.

Adult queens receive mostly royal jelly from nurse bees, possibly with some additional honey^1, 57^. Contaminated food reaches queens through trophallaxis. The contamination might originate from the transfer of active substances through the mandibular and hypopharyngeal glands located in the heads of nurse bees or by the addition of contaminated honey^58^. Although little is known about pesticide contamination of royal jelly, neonicotinoids can be found in the heads of bees were the glands are located^26^. Neonicotinoids have also been detected in brood food^59^ and royal jelly when bees were exposed to contaminated pollen^40^. In addition, neonicotinoids can reduce the size of the royal jelly producing hypopharyngeal glands in nurse bees^60, 61^, thus queens may be indirectly affected by altered food quality or quantity.

It is not clear, whether queens in laboratory assays or in the social context of a colony are exposed to the same dosages of pesticides as worker bees. In a honeybee colony, the queen may be shielded from harmful agents like pesticides by the attending workers. Transmission experiments suggest that behavioural patterns are in place to protect the queen from viral infection by symptomatic bees. Either queens avoid direct contact with or uptake of food from diseased workers, or diseased workers try to avoid interaction with the queen^62^. It is not known, whether similar behavioural mechanisms may exist in a colony that prevent or reduce exposure of queens to pesticides.

To estimate the level of exposure to queens, the detoxification of neonicotinoids has also to be taken into account. In case queens failed to fully clear ingested pesticides from their bodies, the persistence of even small daily intakes could eventually accumulate to harmful or even lethal levels over time. Indeed, the lethality of the neonicotinoid imidacloprid to worker bees, ants or termites appears to be dependent on the duration of exposure: the longer the exposure time, the less amount of pesticide is needed to kill the worker^63^. So far, the temporal dynamics of the decrease of neonicotinoid residues has only been studied in worker bees^26^. To our knowledge, no data are available concerning the ability of queens to detoxify pesticides.

A honeybee colony represents an environment with a high chance for the spread of infections, because of its highly organised social structure and crowded population density^64^. In honeybees, transmission of viruses can occur horizontally among workers, or vertically, from the queen to her offspring. In queens, numerous viruses have been detected, e.g. DWV, chronic bee paralysis virus (CBPV), black queen cell virus, Kashmir bee virus, and sacbrood virus^64^. In a recent sanitary survey in Belgium, 75% of the eggs tested had at least one virus present^65^. It appears plausible that a queen with a weakened immune defence may be prone to infections, which she can transmit vertically to her offspring.

In honeybees, like in other insect species, a maternal immune experience can be transmitted to the progeny. This so called trans-generational immune priming has been demonstrated to have a positive impact on offspring resistance and survival of infections^66, 67^. Whether a queen with weakened immune defence can still sufficiently protect her offspring via these mechanisms has yet to be investigated. Since a queen lays between 175.000 or 200.000 eggs annually^1^, the health status of a queen is highly relevant for the overall colony health.

A young queen has to master several challenges in the early phase of her life. She has to fight her competitive sisters, perform risky nuptial flights, successfully mate with a sufficient number of drones, and start laying fertilised eggs in an adequate number and quality to be accepted by the colony^1^. Especially when the queen gets into contact with drones on her nuptial flights, she is potentially confronted with an additional load of pathogens^68^. We do not know whether the immunosuppressive effect of neonicotinoids may affect the ability of a queen to accomplish these tasks. The impact on the health and fitness of a queen may depend on the phase of life and the duration of the exposure to pesticides. Exposure to neonicotinoid pesticides during larval development of queens can have severe effects on her performance later in life: clothianidin and thiamethoxam treatment during the larval phase affects ovary size and reduces the number and quality of stored spermatozoa within queen spermathecae, which results in reduced egg laying success^5^. In our study, we analysed the immunocompetence of newly hatched queens after only seven days of exposure to neonicotinoids. However, in the field, queens may be exposed to pesticides for several months, including the winter season^69^. Whether queens are susceptible to neonicotinoids during all phases of their life, or whether the immunosuppressive effects are persistent or reversible has yet to be determined.

Immune suppression by pesticides like neonicotinoids opens the way to the spread and abundance of pathogens and parasites, which are the proximate mortality factors of honeybee colonies^11, 36^. Infestation with *Varroa destructor*, the suspected main cause for colony losses, was shown to be promoted by exposing colonies to neonicotinoid treated crops^70^. This study on the immunocompetence of queens complements previous, similar findings on the immunosuppressive effects of neonicotinoids on worker bees^41, 47^. Currently, regulatory requirements for evaluating the safety of pesticides to honeybees do not consider effects on the immune defence on workers, drones, or queens^71^. Given the key importance of queens to colony health and survival, the general lack of knowledge concerning both lethal and sub-lethal effects of pesticides on queen physiology is alarming. Our findings highlight the vulnerability of honeybee queens to common neonicotinoid pesticides, and demonstrate the need for future studies to identify relevant measures of queen health and disease susceptibility. Improving the understanding of honeybee immunity could provide new insights into the stress factors and their interactions that threaten honeybee survival and ultimately enable us to design strategies to protect them^11^.

## Material and Methods

### Rearing of Queens

Queens were produced using standard honeybee queen-rearing techniques^72^. Briefly, young grafted larvae were introduced into queenless starter colonies. After 5 days, sealed queen cells were transferred to an incubator (35 °C, 65% humidity; Grumbach, Asslar, Germany). Sister queens from eight different maternal lines were distributed equally among the treatment groups. After emergence, queens were visually inspected and transferred into standard metal cages (8.5 × 6.5 × 4 cm,) together with ten attendant workers from healthy experimental colonies that tested negative for virus infections (DWV, acute bee paralysis virus, CBPV, sac brood virus)^4, 62^.

### Neonicotinoid exposure in laboratory cage experiments

The cages were supplied with water and pollen (collected at the Bee Institute Kirchhain, Germany), and *ad libitum* sugar syrup (Apiinvert, Mannheim, Germany) diluted to a 50% sugar solution (w/v with ambrosia Bienenfutter, Germany and distilled water) in a 5 ml syringe (Carl Roth, Karlsruhe, Germany). Cages were kept in an incubator (Binder, Tuttlingen, Germany; humidity provided by open water jars) in the dark at 33 °C^73^.

Thiacloprid was obtained from Sigma Aldrich (St. Louis, USA; analytical standards, purity 99.9%). A stock solution of 2 mg/ml thiacloprid in acetone was prepared in a glass flask and stored in the dark at room temperature (~15 °C) until use. A sugar solution (50% w/v) was prepared and thiacloprid stock solution was added to reach a final concentration of 100 or 200 µg/l thiacloprid. The final concentration of acetone in the feeding solutions was adjusted to 0.0086% (v:v) in all thiacloprid treatment groups, including the control. Clothianidin (Sigma Aldrich, analytical standards, purity 99.3%) was dissolved in water and added to the sugar solution (50% w/v) to obtain a final concentration of 10, 50, or 200 µg/l clothianidin. The pollen pastry was prepared from pollen collected at the Bee Institute Kirchhain or obtained from Imkereibedarf Bährle (Aschaffenburg, Germany).

Honeybees of each cage (= a queen with ten worker bees) were exposed to one of these concentrations for seven days. During the phase of exposure, queens remained within the group of the attendant bees in the cages. In that way queens were exposed to the dietary pesticide either directly by taking up contaminated food themselves or indirectly via the food (e.g. royal jelly) received from the attending worker bees. The consumption of food was recorded daily. To quantify the food consumption, the syringes containing the sugar solution and the caps containing the pollen were weighed after 24 hours in the cage. Food consumption was calculated by dividing the total amount of food consumed in 24 hours divided by the number of workers in the cage. Three empty cages contained an evaporation control. Dead individuals were removed every 24 or 48 hours (for details see Supplementary Table S2).

Subsequently, the immunocompetence of the queens was evaluated by one of the methods: quantification of hemocytes, antimicrobial activity of the hemolymph, or melanisation response. Any individual queen was used for only one single immune test. Each test was replicated at least three times with at least 14 queens per treatment group (details see below and Supplementary Table S1).

### Hemolymph collection

Queens were anesthetized on ice before hemolymph was collected by inserting a microinjection needle (Hartenstein, Würzburg, Germany) into the proximal abdomen. Any fluid which appeared yellow or brown was discarded and excluded from further analysis as this was likely not hemolymph but gastric fluid^33^.

### Total and differential hemocyte counts

Total hemocyte counts were performed as an indirect measurement of cellular immunocompetence^33^. For total hemocyte counts, 1 µl of hemolymph was transferred to a PCR-tube (Biozym, Hessisch Oldendorf, Germany) containing 3 µl PBS (pH 7.4; Sigma Aldrich, St. Louis, USA) and 1 µl of DAPI-staining solution (4′,6-diamidino-2-phenylindole; 1:100 dilution, lifetechnologies, Carlsbad, California, USA). Immediately after collection, the diluted hemolymph solution was transferred to a Bürker counting chamber (Carl Roth, Karlsruhe, Germany), where hemocytes were counted (average of five squares per queen) under a phase contrast/fluorescent microscope (Leica DMIL, Leica camera DFC 420 C). To verify the cellular character of the observed structures, DAPI staining was used as counterstaining of nuclear DNA^47^. Each experiment was repeated five times with 14 to 19 queens per treatment group.

To analyze whether exposure to pesticides affects the composition of the hemocyte population, differential hemocyte counts were conducted. The hemolymph (3 µl) was placed on sterile glass cover slips in a cell culture 24-multiwell plate (Sigma Aldrich) with distilled water in the spaces between the wells. The multiwell plate was placed in the incubator for 4 hours at 33 °C. Subsequently, the cells were fixed in formaldehyde solution (4% formaldehyde in PBS, Carl Roth) over night at 4 °C, rinsed three times in PBS and permeabilized in PBS + 0.2% Tween-20 (Sigma Aldrich) for 10 min. To visualize the cytoskeleton and the nuclei, cells were stained with Alexa 555-conjugated phalloidin at 1:200 (Invitrogen, Carlsbad, USA) and DAPI at 0.3 µM for 30 min, washed with PBS and mounted in Vectashield (VWR International, Darmstadt, Germany). Queen hemocytes were visualized using a confocal microscope (Zeiss LSM 700, Jena, Germany) or observed under the phase contrast/fluorescent microscope. Following the description and classification of Negri *et al*.^50^, we classified the queen hemocytes using morphological characteristics (eight to nine queens per treatment group, 351 to 530 cells counted per individual; for details see Supplementary Table S1).

### Melanisation

To provoke a melanisation response, a nylon filament was partly inserted into the abdomen of a queen, thus mimicking the behaviour of *Varroa destructor* as previously described by Brandt *et al*.^47^. The strength of the immune reaction was measured by the degree of melanisation on the filament. Briefly, a nylon fishing line (0.2 mm diameter, Nexos, Naila, Germany) was cut into approximately 2.5 mm long segments and sterilized in 100% pure ethanol (Roth, Karlsruhe, Germany). Queens were anesthetized on ice, and the nylon filament was implanted in the abdomen through the intersegmental membrane between the 3^rd^ and 4^th^ tergum^33, 74^. After implantation, queens were transferred to a 2 ml microcentrifuge tube (Eppendorf, Hamburg, Germany) with holes poked through cap and sidewalls. After approx. four hours, the nylon filament was extracted, fixed in formaldehyde solution for at least 1 hour, rinsed three times in PBS, and subsequently mounted in glycerol (85%, Carl Roth). Each experiment was repeated four times (17 to 19 queens per treatment group). Three pictures per explant were taken at different focal depths. The mean grey value per filament served as a measure of melanisation and was quantified for the inserted portion of the filament using image analysis software^73^. The mean grey value of an non-implanted filament that served as background value was subtracted from the mean grey value of the implanted filaments^47^. Each treatment group (for details see Supplementary 15 to 20 queens per treatment group (for details see Supplementary Table S1).

### Inhibition-Zone Assay

For inhibition zones assays, queens were exposed to neonicotinoids for seven days. On day six of the exposure, the immune system was challenged by the injection of 1 µl of heat-inactivated *Escherichia coli* (grown to OD 0.5). As previously described^47^, hemolymph was collected and stored at −20 °C until the assay was conducted. Antibacterial test plates (ø 9 cm) were prepared by adding 0.8 ml of live *Micrococcus luteus* bacteria suspension (OD 0.5) to 150 ml of sterile broth medium (48 °C, 1.5 g Agar No. 1, Oxoid; 3.75 g nutrient broth, Applichem). Per test plate, five holes (ø1 mm) were punched into the medium and 1 µl of hemolymph solution was added to each one. The plates were incubated at 38 °C overnight and the diameter of inhibition zones was measured. Each experiment was repeated three times with 14 to 16 queens per treatment group.

### Hypopharyngeal gland size of attendant bees

In order to determine whether caged queens received sufficient food, the HPG size of attending worker bees was measured. To obtain worker bees of defined age, single frames of late stage capped brood were brought to the laboratory and incubated in the dark at 33 °C (humidity provided by open water jars). The frames with worker brood were collected from two institute colonies, which were regularly inspected for symptoms of diseases. Newly emerged bees (≤24 h) were collected, colour marked and returned to the colony of origin. On day seven after emergence, the marked bees were re-collected and placed into the cages containing the queens. After seven days of exposure to neonicotinoids in the laboratory, the workers were immobilized on ice and the HPGs were dissected in ice cold phosphate buffered saline (PBS, pH 7.4). The specimens were fixed in formaldehyde (4% in PBS, Carl Roth), rinsed three times in PBS and mounted in Aquapolymount (Polysciences, Eppelheim; Germany). Three pictures of each gland (only one gland per bee) were photographed using a Leica phase contrast/fluorescence microscope and image capturing software (Leica, LASV4.4, Wetzlar, Germany). To measure the size of the glands, the diameter of 15 acini per bee was measured with ImageJ software (ImageJ 1.490; Image Processing and Analysis in Java, http://rsb.info.nih.gov/ij/index.html)^75^. The experiment was repeated three times with 20 to 33 individuals per treatment group (for details see Supplementary Table S2).

### Statistical methods

Total hemocyte counts, melanisation/mean grey values, and mean diameters of inhibition zones were not normally distributed, and hence non-parametric statistics were used. Each immunocompetence measure was compared between groups treated with neonicotinoids and untreated control queens using KWT followed by post-hoc pair wise comparisons with Mann–Whitney U tests (MWU). Proportions of cell types were arcsin-transformed before performing statistical testing. The probability levels inferior to 0.05 were corrected for multiple testing according to the Holm’s sequential Bonferroni procedure^76^. All statistical tests were run with the computer program SPSS for Windows (v. 20).

## Electronic supplementary material

Supplementary information.
