# Supplementary material for: Immunosuppression in Honeybee Queens by the Neonicotinoids Thiacloprid and Clothianidin
Source: Sci Rep. 2017 Jul 5;7:4673. doi: 10.1038/s41598-017-04734-1 (PMC5498664; doi:10.1038/s41598-017-04734-1)
Supplement: Supplementary file 1 — Supplementary information. [file 41598_2017_4734_MOESM1_ESM.pdf]

SREP-16-48251-A

Supplementary information

Immunosuppression in Honeybee Queens by the Neonicotinoids Thiacloprid and Clothianidin

Annely Brandt[1*], Katharina Grikscheit[2], Reinhold Siede[1], Robert Grosse[2], Marina Doris Meixner[1], Ralph Büchler[1]

Supplementary table T1 Statistic description of immune related parameters

**Total hemocyte counts**	**Number of queens**	**Median (hemocytes/µl)**	**KWT** p-value	**MWU*** (control vs. treatment)
control	19	4250	0.011	
thiacloprid 200 µg/l	16	2500		0.022
thiacloprid 2000 µg/l	19	2000		0.024
control	19	4250	0.006	
clothianidin 10 µg/l	15	2250		0.015
clothianidin 50 µg/l	14	2000		0.014
**Differential hemocyte counts**		**Median number of cells (%)**	**KWT** p-value	**MWU*** (control vs. treatment)
**Cell type W1**				
control	9	3.356	0.0005	
thiacloprid 200 µg/l	9	1.980		0.002
clothianidin 10 µg/l	8	0.394		0.034
**Cell type W2**				
control	9	14.75	0.0384	
thiacloprid 200 µg/l	9	7.97		
clothianidin 10 µg/l	8	6.84		0.060
**Cell type W3**				
control	9	65.78	> 0.05	
thiacloprid 200 µg/l	9	71.79		
clothianidin 10 µg/l	8	83.07		
**Cell type W4**				
control	9	12.81	> 0.05	
thiacloprid 200 µg/l	9	10.39		
clothianidin 10 µg/l	8	9.16		
**Encapsulation response**	**n**	**Median grey value (%)**	**KWT** p-value	**MWU*** (control vs. treatment)
control	20	23.83	0.001	
thiacloprid 200 µg/l	17	10		0.016
thiacloprid 2000 µg/l	15	5.5		0.003
control	20	23.83	< 0.0001	
clothianidin 10 µg/l	19	7.5		< 0.0001
clothianidin 50 µg/l	19	7		0.002
**Antimicrobial activity**	**n**	**Median inhibition zone (Ø mm)**	**KWT** p-value	**MWU** (control vs. treatment)
control	15	19.37	0.002	
thiacloprid 200 µg/l	16	17.25		0.008
thiacloprid 2000 µg/l	15	16.42		0.003
control	15	19.37	0.001	
clothianidin 10 µg/l	14	17.26		< 0.001
clothianidin 50 µg/l	14	16.54		< 0.001

* p-value, two sided, Holm-Bonferroni corrected
KWT - Kruskal Wallis test
MWT - Mann–Whitney U test

Supplementary table T2 Statistic description of lethality, food consumption and hypopharyngeal gland (HPG) size

Percentage of dead queens	n	%	χ^2-test
control	51	9.08	0.458
thiacloprid 200 µg/l	49	10.20	
thiacloprid 2000 µg/l	49	4.08	
control	50	9.08	0.115
clothianidin 10 µg/l	45	8.88	
clothianidin 50 µg/l	46	15.21	
clothianidin 200 µg/l	25	28	

Percentage of dead workers	(n)	%	χ^2-test
control	510	9.08	0.456
thiacloprid 200 µg/l	490	10.20	
thiacloprid 2000 µg/l	490	4.08	
control	500	9.08	< 0.0001
clothianidin 10 µg/l	450	8.88	
clothianidin 50 µg/l	460	15.21	
clothianidin 200 µg/l	250	28	

Consumption of sugar solution	n	Average (g/worker/day)	KWT p-value
control	280	0.288	0.402
thiacloprid 200 µg/l	280	0.282	
thiacloprid 2000 µg/l	280	0.256	
control	280	0.288	0.420
clothianidin 10 µg/l	250	0.263	
clothianidin 50 µg/l	240	0.272	
clothianidin 200 µg/l	240	0.304	

Consumption of pollen	n	Average (g/worker/day)	KWT p-value
control	280	0.028	0.478
thiacloprid 200 µg/l	280	0.023	
thiacloprid 2000 µg/l	280	0.024	
control	280	0.028	0.470
clothianidin 10 µg/l	250	0.024	
clothianidin 50 µg/l	240	0.025	
clothianidin 200 µg/l	240	0.025	

Acinus diameter of HPGs	n	Average (g/worker/day)	KWT p-value	MWU* (control vs. treatment)
control	27	122	0.286	
thiacloprid 200 µg/l	23	115		
thiacloprid 2000 µg/l	20	127		
control	27	122	0.025	
clothianidin 10 µg/l	25	123		
clothianidin 50 µg/l	22	132		
clothianidin 200 µg/l	33	113		
control vs. clothianidin 200 µg/l				0.003

* p-value, two sided, Bonferroni corrected
n = number of individuals
KWT - Kruskal Wallis test
MWT - Mann–Whitney U test

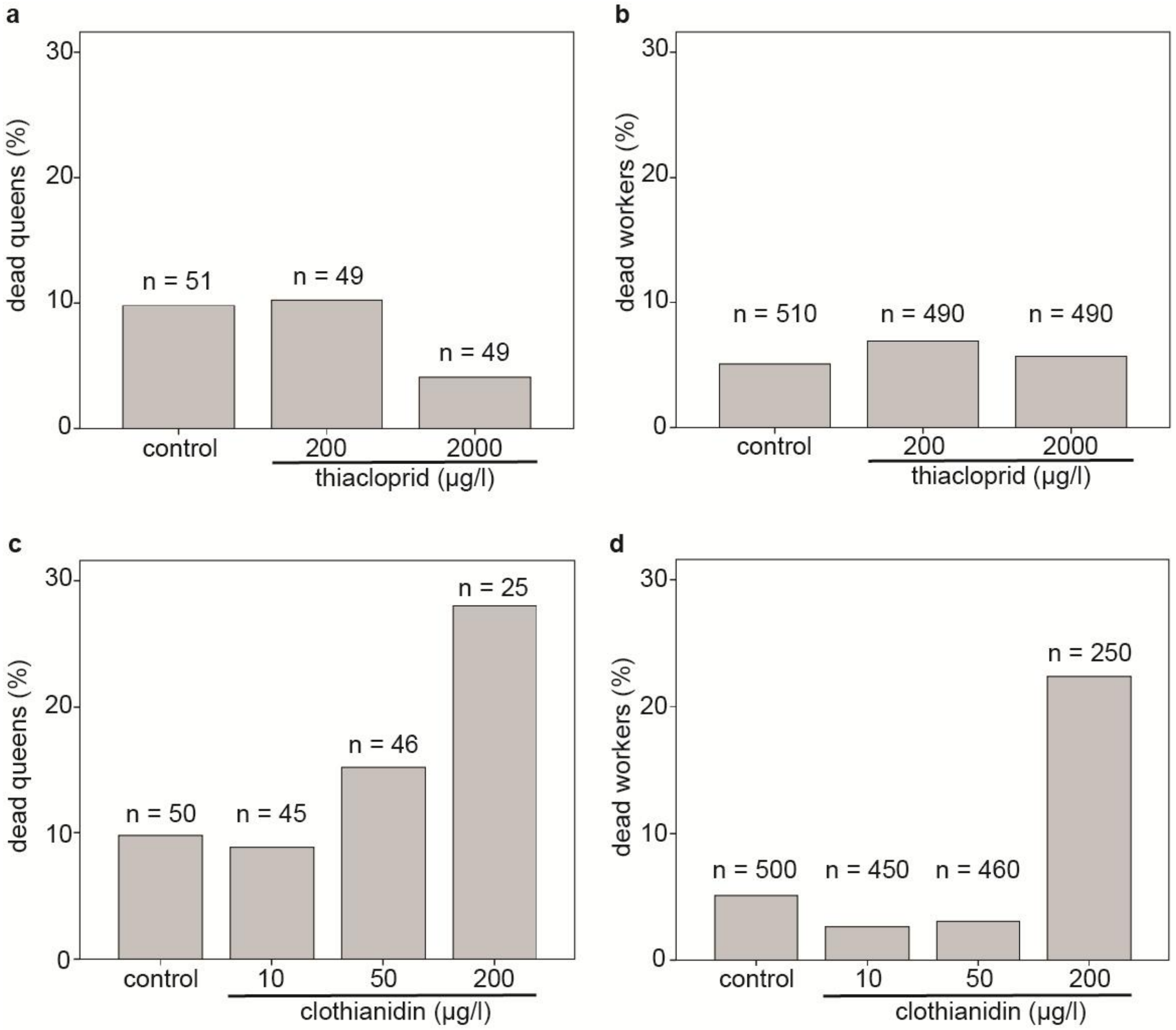

Supplementary Fig. S1 Percentage of dead queens (a, c) or attendand workers (b, d) after 7 days of exposure. The number of individuals (n) for each treamtment group incorporated into this analysis is depicted above the corresponding bar. There were no statistically significant differences between thiacloprid exposed queens (**a**) or workers (**b**) or clothianidin exposed queens and the corresponding controls **(c**; X^2-test, $p > 0.05$). However, exposure to clothianidin had an effect on worker bee survival (X^2-test, $p < 0.001$).

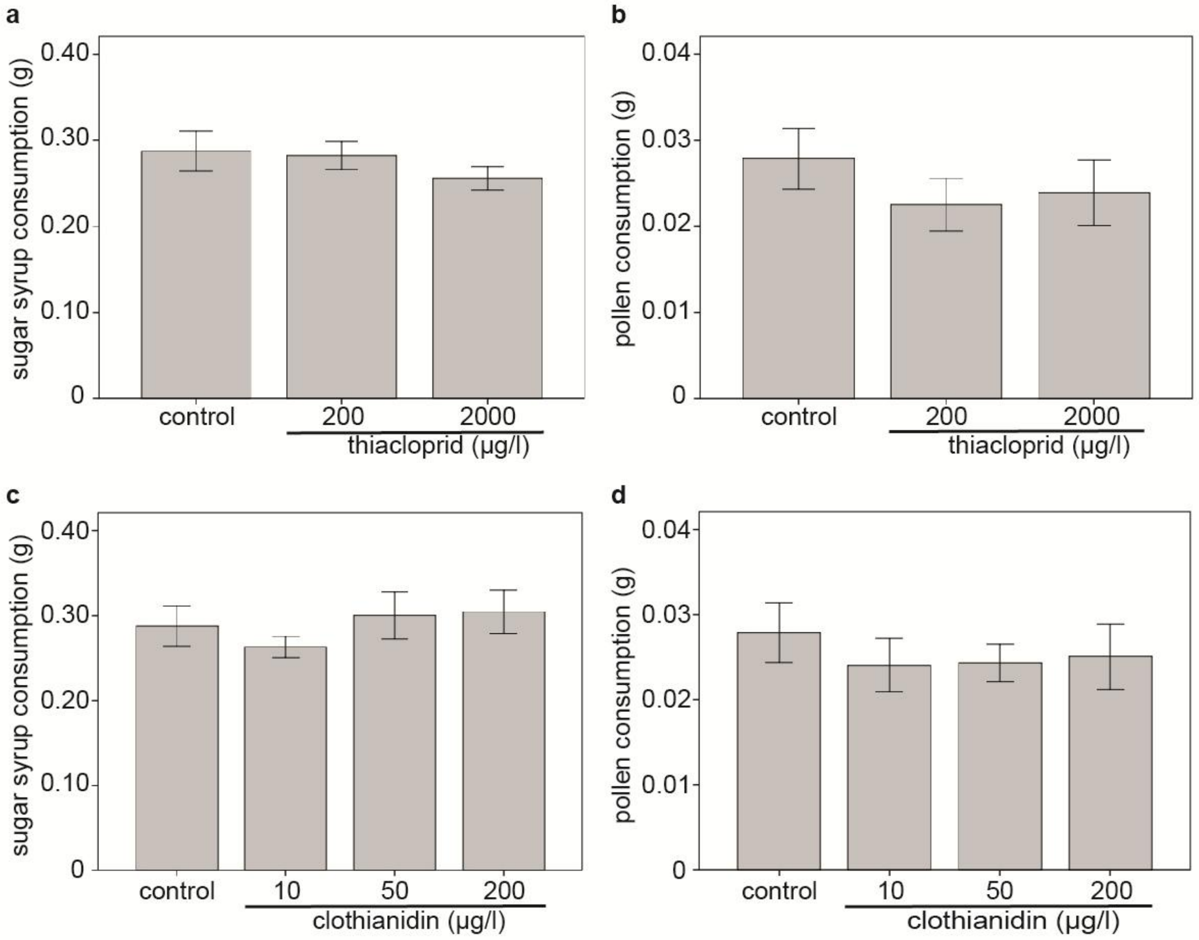

Supplementary Fig. S2 Sugar sollution or pollen consumption. The consumption of sugar solution (**a**, **c**) or pollen (**b**, **d**) was recorded per worker bee per day. There were no significant differences between the treatment groups (KWT, $p > 0.05$). Error bars indicate standard errors.

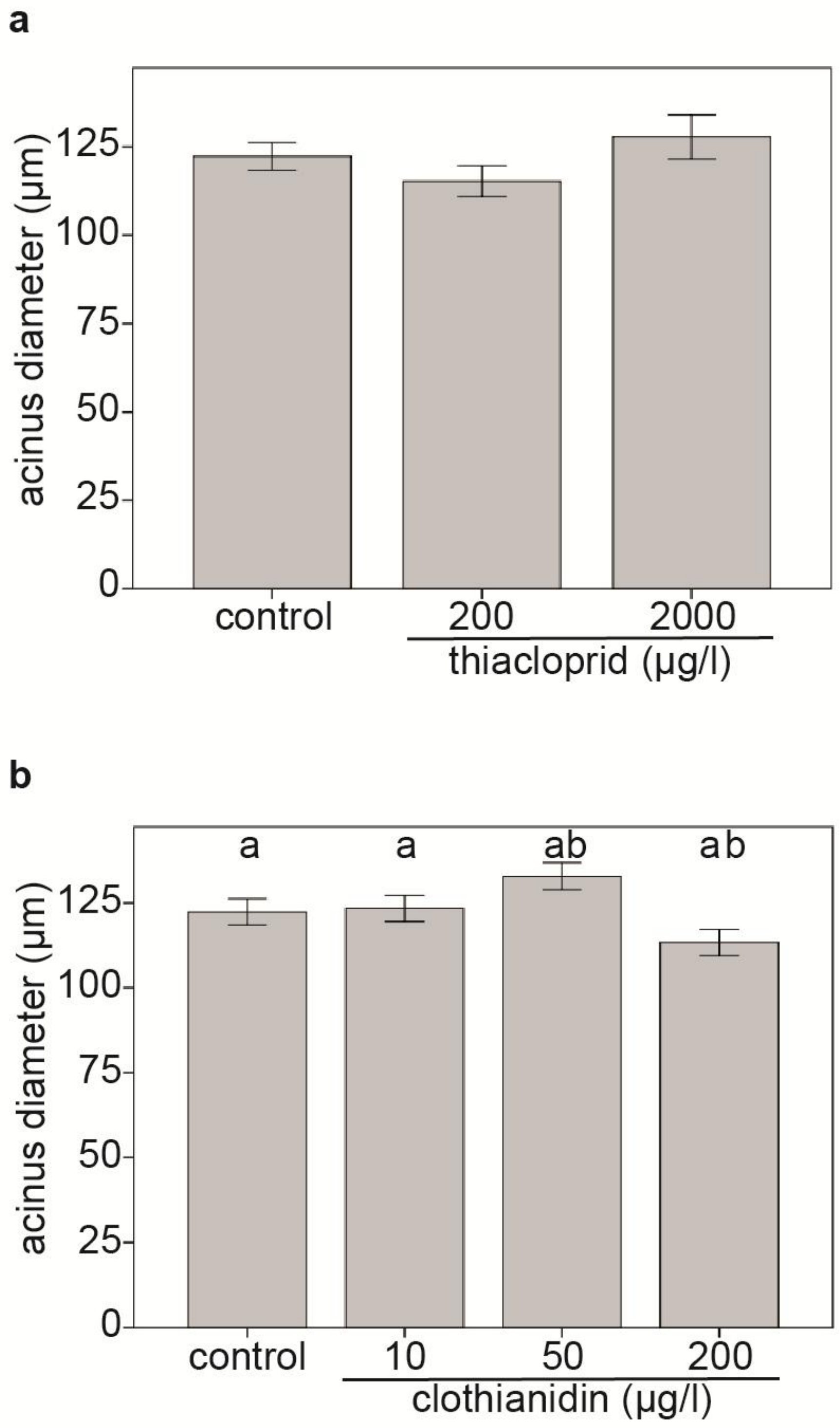

Supplementary Fig. S3 Hypopharyngeal gland size of attendant bees. The acini diameter of age defined worker bees was not affetced after exposure to thiacloprid (**a**; 200 or 2000 µg/l; KWT $p > 0.05$) or clothianidin (**b**; 10, 50, or 200 µg/l) for seven days when compared to controls. Only clothiandin 50 µg/l was significantly different from 200 µg/l group (KWT $p = 0.25$; MWT, $p = 0.003$). Significant differences indicated with letters, error bars indicate standard errors.
